# Muscle Fatigue Affects the Interpolated Twitch Technique When Assessed Using Electrically-Induced Contractions in Human and Rat Muscles

**DOI:** 10.3389/fphys.2016.00252

**Published:** 2016-06-28

**Authors:** Daria Neyroud, Arthur J. Cheng, Nicolas Bourdillon, Bengt Kayser, Nicolas Place, Håkan Westerblad

**Affiliations:** ^1^Institute of Sport Sciences, Faculty of Biology and Medicine, University of LausanneLausanne, Switzerland; ^2^Department of Physiology, Faculty of Biology and Medicine, University of LausanneLausanne, Switzerland; ^3^Department of Physiology and Pharmacology, Karolinska InstitutetStockholm, Sweden

**Keywords:** voluntary activation, contractile properties, interpolated twitch, M-wave, central fatigue

## Abstract

The interpolated twitch technique (ITT) is the gold standard to assess voluntary activation and central fatigue. Yet, its validity has been questioned. Here we studied how peripheral fatigue can affect the ITT. Repeated contractions at submaximal frequencies were produced by supramaximal electrical stimulations of the human *adductor pollicis* muscle *in vivo* and of isolated rat *soleus* fiber bundles; an extra stimulation pulse was given during contractions to induce a superimposed twitch. Human muscles fatigued by repeated 30-Hz stimulation trains (3 s on–1 s off) showed an ~80% reduction in the superimposed twitch force accompanied by a severely reduced EMG response (M-wave amplitude), which implies action potential failure. Subsequent experiments combined a less intense stimulation protocol (1.5 s on–3 s off) with ischemia to cause muscle fatigue, but which preserved M-wave amplitude. However, the superimposed twitch force still decreased markedly more than the potentiated twitch force; with ITT this would reflect increased “voluntary activation.” In contrast, the superimposed twitch force was relatively spared when a similar protocol was performed in rat *soleus* bundles. Force relaxation was slowed by >150% in fatigued human muscles, whereas it was unchanged in rat *soleus* bundles. Accordingly, results similar to those in the human muscle were obtained when relaxation was slowed by cooling the rat *soleus* muscles. In conclusion, our data demonstrate that muscle fatigue can confound the quantification of central fatigue using the ITT.

## Introduction

The interpolated twitch technique (ITT) developed by Merton (1954) is considered the gold standard to evaluate non-invasively the ability to maximally activate motor units in healthy and clinical populations (Gandevia, 2001; Millet et al., 2012). It consists of electrically (or magnetically) stimulating a nerve trunk or axonal terminal branches during a maximal voluntary contraction. An increase in force elicited by the superimposed stimulation highlights a deficit in voluntary activation. As such, ITT is the most commonly used method to assess central (neural) alterations during exercise (Gandevia, 2001; Millet et al., 2012). Despite its wide use, the validity of ITT to measure the extent of voluntary activation is still debated (de Haan et al., 2009; Taylor, 2009; Contessa et al., 2016).

Place et al. (2008) challenged the validity of ITT for central fatigue assessment with an *in vitro* model of isolated single fast-twitch fibers of mouse *flexor digitorum brevis* (FDB) muscles. These fibers were fatigued by repeated tetani induced by direct electrical stimulation while introducing an extra stimulation pulse during contractions to mimic the ITT. The results showed a relative increase in the interpolated twitch amplitude with fatigue. Such a result, had it been observed in humans during voluntary contraction, would have been interpreted as central fatigue, a phenomenon that is obviously impossible in isolated fibers. Subsequently, Gandevia et al. (2013) performed similar experiments with electrically-evoked tetanic contractions with an extra stimulation pulse during contractions to simulate the ITT in human *adductor pollicis* muscle. In contrast to the results of Place et al. (2008), they observed a *reduction* in the interpolated twitch force with fatigue development.

In the present study we addressed two possible reasons to the above described conflicting results. First, the mouse FDB fibers were fatigued by repeated brief contractions, whereas the human *adductor pollicis* muscle was exposed to 1 min of continuous stimulation, which might decrease the interpolated twitch force due to impaired action potential generation and propagation (Bigland-Ritchie et al., 1979; Duchateau and Hainaut, 1985; Lännergren and Westerblad, 1987; Clausen and Nielsen, 2007; Place, 2008). Indeed, in Gandevia et al. (2013) the decrease in interpolated twitch force in the fatigued state was accompanied by a reduced compound muscle action potential (M wave) amplitude. Second, the conflicting results might reflect the use of *fast-twitch* mouse FDB fibers (Calderón et al., 2009) by Place et al. (2008), whereas Gandevia et al. (2013) used the largely *slow-twitch* human *adductor pollicis* muscle (Johnson et al., 1973). To address these two points, we fatigued human *adductor pollicis* muscles with intermittent contractions and replaced mouse FDB fibers with slow-twitch rat *soleus* fibers (Mizunoya et al., 2014; Soukup and Diallo, 2015).

## Materials and methods

### Ethical approval

For human experiments, all participants gave their written informed consent before participation. The experimental protocol was approved by the Research Ethics Committees of the Geneva (13–107) and Vaud cantons (128/14) and were in agreement with the Declaration of Helsinki. Twenty-four healthy subjects participated in the study (8 women and 16 men, 28 ± 6 years old).

All animal experiments complied with the Swedish Animal Welfare Act, the Swedish Welfare Ordinance, and applicable regulations and recommendations from Swedish authorities. The study was approved by the Stockholm North Ethical Committee on Animal Experiments. Five 6–8 week old male Wistar rats were killed by placing them in a chamber filled with CO_2_.

### Human experiments

#### Experimental setup

Subjects sat on a chair that was adjustable for height, with their right forearm resting in a custom-made mold and the elbow and shoulder angles set to 90° in the sagittal axis. Two straps tightly secured the forearm (10 cm above the wrist and 5 cm below the elbow crease) to the ergometer. The thumb was adjusted to an angle allowing optimal force development and its first phalanx positioned on a support connected to the strain gauge (Z8 500 N, sensitivity 2 mV/V and 0.0083 V/N; HBM, Darmstadt, Germany). Force signals were recorded at 1 kHz using an analog-digital conversion system (MP150; BIOPAC, Goleta, CA, USA).

Transcutaneous electrical stimulation of the ulnar nerve was delivered by a high-voltage (400 V maximum) constant-current stimulator (DS7AH; Digitimer, Hertfordshire, UK) driven by a stimulation train generator (MP150; BIOPAC, Goleta, CA). The cathode and anode (4-mm plug bar-handle stimulator, SPES Medica, Genova, Italy) were located over the ulnar nerve anteriorly and just proximal to the wrist (Neyroud et al., 2013). We used 0.5-ms rectangular-wave pulses and a current intensity set to 120% of the intensity producing the maximal twitch force and compound action potential (M-wave) amplitude.

The surface electromyographic (EMG) activity of the *adductor pollicis* muscle was recorded with a pair of circular silver chloride (Ag/AgCl, 1-cm recording diameter) self-adhesive electrodes (Meditrace 100, Tyco, Canada), which were cut to obtain an inter-electrode distance (center-to-center) of 1.5 cm and positioned over the muscle belly. A reference electrode was placed over a proximal radius protuberance. Low resistance between the two electrodes was obtained by cleaning and lightly abrading the skin. EMG signals were amplified with a gain of 1000, digitized at a sampling frequency of 2 kHz, filtered with a bandwidth frequency between 10 and 500 Hz and recorded by an analog-digital conversion system (MP150; BIOPAC, Goleta, CA, USA). EMG as well as force signals were stored and analyzed offline with commercial software (Acqknowledge, BIOPAC Systems, Goleta, CA, USA).

#### Experimental protocols

Initially 1-s current trains (separated by ~10 s) were evoked, at 10, 15, 20, 30, 50, 80, and 100 Hz, in a counterbalanced order between subjects, to determine the force-frequency relationship in the rested state. Thereafter one of three different fatiguing protocols (*Exp1, Exp2, or Exp3*) was used. Finally, one 1-s contraction at 100 Hz was produced ~1 min after the end of fatiguing stimulation.

In the initial set of experiments (*Exp1*), fatigue was induced by 28 contractions evoked at 30 Hz with a duty cycle of 3 s contractions interspersed with 1-s resting periods. To mimic ITT, an additional electrical pulse (i.e., superimposed) was sent 1.5 s into every third contraction and 15 ms (see Gandevia et al., 2013) were separating this additional pulse from the previous regular pulse. An electrical pulse was also delivered 0.8 s after the end of every third contraction to measure the potentiated peak twitch force and the associated M-wave properties.

In the next set of experiments (*Exp2*) we used a lower duty cycle (1.5 s contraction, 3 s rest period) than in *Exp1*. During each of these contractions, an additional electrical stimulus was delivered 1 s into the contraction with 15 ms separating this additional pulse from the previous regular pulse. An electrical pulse was also delivered 1.5 s after the end of each evoked contraction (i.e., potentiated twitch). As pilot experiments showed very limited fatigue with this duty cycle, stimulation was performed under ischemia induced by inflating to 250 mmHg a 13 × 85-cm cuff (SC12D, Hokanson, Bellevue, USA) wrapped around the upper arm, fully occluding the circulation (Taylor et al., 2000). The cuff pressure was briefly released between the pre-fatigue force-frequency relationship and the beginning of the fatiguing task to limit the duration of occlusion. The cuff was then kept inflated throughout the fatiguing task and until the 100-Hz stimulation train delivered post-fatigue.

In the final set of experiments (*Exp3*) we used the same procedures as in *Exp2* but contractions were produced at 20 Hz instead of 30 Hz since the latter stimulation frequency can produce close to maximal forces, whereas reducing the stimulation frequency would provide more room for a potential superimposed twitch to increase.

#### Data analysis

For all parameters, the different values obtained throughout the course of the fatiguing task are expressed as a percentage of their value obtained during the first tetanus.

##### Force

Every third evoked contraction of the fatiguing task was considered for analysis. For these contractions, amplitudes of potentiated and superimposed twitch forces as well as the force level just before the superimposed twitch (referred to as tetanic force from now on) were measured. The half-relaxation time (HRT) was measured in the first and last fatiguing contractions as the time from the end of stimulation until force had declined to 50% of the tetanic force. For the force-frequency relationship, the mean force over a 0.5-s window was measured at each frequency and expressed as a percentage of the force produced by the 100-Hz stimulation train.

##### EMG

The M waves associated with the superimposed electrical stimulus (superimposed M-wave) were analyzed. However, during fatiguing stimulation, the high stimulation frequency led to a truncated M-wave in between two stimulation artifacts in some participants and therefore peak-to-peak M-wave amplitude and duration could not be consistently measured. Therefore, the M-wave amplitude was quantified as the amplitude of the first peak of the M-wave (referred to as M-wave amplitude from now on, see Rodriguez-Falces and Place, 2016). The latency of the M-wave (reflecting action potential propagation along both the axons and muscle fibers) was quantified as the time between the stimulation artifact to the first peak of the M-wave (Rodriguez-Falces and Place, 2013).

### Animal experiments

#### Experimental protocol

Whole *soleus* muscles were removed from the hindlimbs of the rats and mechanically dissected into bundles of ~3–5 fibers with intact tendons. Aluminium T-clips were attached to the tendons and the fiber bundles were mounted in a chamber between a force transducer (Akers 801, Kronex Technologies, Oakland, California, USA) and an adjustable holder. Fibers were electrically stimulated with 0.5-ms current pulses via platinum electrodes placed on both sides of the fiber bundle. Fiber length was adjusted to achieve maximal tetanic force. The fibers were continuously superfused at room temperature (23°C) with a standard Tyrode solution (in mM): 121 NaCl, 5.0 KCl, 1.8 CaCl_2_, 0.5 MgCl_2_, 0.4 NaH_2_PO_4_, 24.0 NaHCO_3_, 0.1 EDTA, and 5.5 glucose. The solution was bubbled with 95% O_2_–5% CO_2_, giving a bath pH of 7.4. Fatigue was induced at 23°C with repeated 3-s contractions at a frequency giving close to 70% of maximum tetanic force. These contractions were produced every 4 s until force decreased to 50% of the initial value. During each of these contractions, an additional electrical stimulus was delivered 2.5 s into the contraction with 10 ms separating this additional pulse from the previous regular pulse. An electrical doublet pulse, with a 10-ms inter-pulse duration, was also delivered 1 s after the first and last contraction of the repeated stimulation protocol.

In another set of experiments designed to assess the effect of contractile slowing on ITT, force-frequency relationships were obtained at two temperatures (23 and 18°C). Three second duration tetani were evoked at 1-min intervals at 10, 15, 20, 30, 40, and 50 Hz at 18°C, and also at 70 and 100 Hz at 23°C. At each frequency, an additional electrical stimulus was delivered 2.5 s into the contraction with 10 ms separating this additional pulse from the previous regular pulse. As for the fatiguing experiments, an electrical doublet pulse with a 10 ms interpulse interval was delivered 1 s after each tetanus (i.e., the potentiated twitch). Peak forces were measured for tetani, and for the superimposed and potentiated twitches. The superimposed twitch force was measured as the force prior to the additional electrical stimulus up to peak force following the superimposed stimulus. HRT was measured as the time from the end of stimulation until force had declined to 50% of the tetanic force.

### Statistical analysis

For human experiments, depending on the outcome of the Shapiro-Wilk normality test, one-way or Friedman repeated measures ANOVAs [time (tetanus 1, 4, 7, 10, 13, 16, 19, 22, 25, and 28)] were performed for all parameters. When significant differences were found, Dunnett's *post hoc* was applied to test for differences from initial values. Unpaired *t*-tests were performed to compare differences in relative changes between *Exp1* and *Exp2*, and between *Exp2* and *Exp3* as well as in initial values between *Exp2* and *Exp3*. A paired *t*-test was used to compare the forces evoked by the 20 and 30-Hz stimulation trains before the fatiguing task. For the rat *soleus* fiber experiments, paired *t*-tests were used to compare values obtained during the first and last contraction of the fatiguing stimulation, as well as to compare values obtained at each stimulation frequency between 18°C vs. 23°C. One-way ANOVA was performed to detect differences in the tetanic HRT between human *adductor pollicis* muscle and rat *soleus* fiber bundles. The α-level for statistical significance was set at *p* < 0.05. Sigmaplot software for Windows (version 11, Systat, Chicago, IL) was used for all statistical analyses. Data are reported as mean ± SD.

## Results

### Human experiments

Fourteen participants took part in *Exp1* (25 ± 6 years), 8 in *Exp2* (31 ± 6 years), and 6 in *Exp3* (30 ± 3 years). Table 1 presents force and EMG data from the first tetanic contraction of the three different fatiguing protocols.

**Table 1:** **Initial values of force and electromyographic (EMG) parameters**.

	***Exp1***	***Exp2***	***Exp3***
Superimposed twitch, N	3.0 ± 1.3	3.3 ± 1.3	4.8 ± 1.3
M-wave amplitude, mV	1.6 ± 1.5	1.5 ± 1.5	3.9 ± 1.5
M-wave latency, ms	6.7 ± 1.1	6.5 ± 0.5	6.6 ± 0.4
Tetanic force, N	59.7 ± 17.8	62.2 ± 18.8	52.9 ± 13.8
Tetanic HRT, ms	88 ± 13	82 ± 7	81 ± 9
Potentiated twitch, N	9.2 ± 2.7	10.7 ± 2.9	10.5 ± 3.0

*HRT = half relaxation time. Data are expressed as mean ± SD; n = 14 in Exp1, 8 in Exp2, and 6 in Exp3*.

#### Exp1

**Figure 1:**
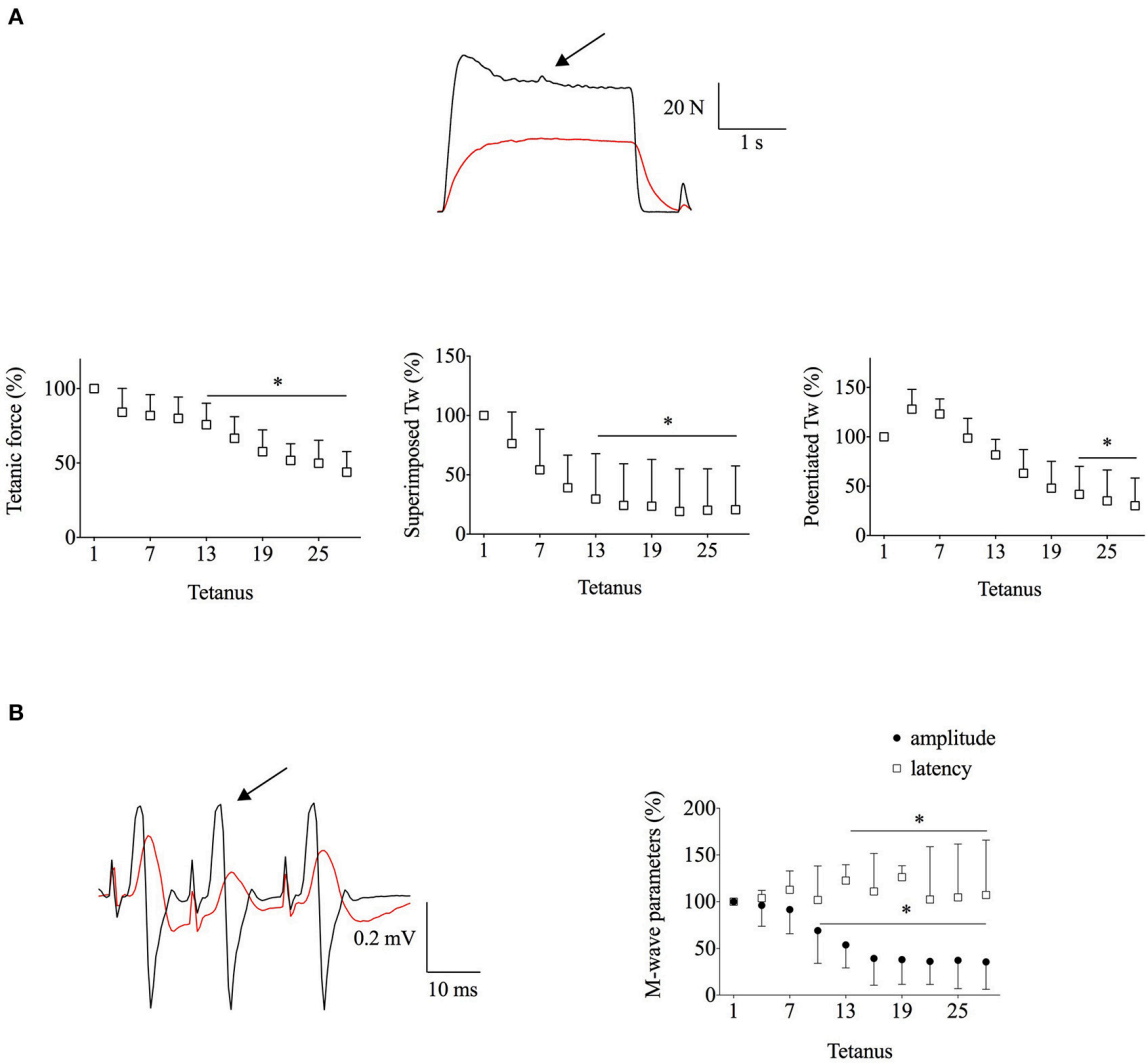
**The intense stimulation in ***Exp1*** resulted in major decreases in force and M-wave amplitudes**. Human *adductor pollicis* muscles were stimulated at 30 Hz, 3 s on–1 s off. **(A)**, the upper part shows representative force records of the first and last fatiguing contractions and the lower part mean data of tetanic and peak twitch (Tw) forces; expanded superimposed twitches are shown above the tetanic force records. **(B)**, EMG records (left) and mean M-wave amplitude and latency (right). Black and red lines in original records correspond to the first and the last tetanus; respectively; arrow indicates the extra stimulation. Data are mean ± SD expressed in percentage of the initial value (*n* = 10). ^*^p < 0.05 shows significant difference from the first tetanus (one-way repeated measures ANOVA).

Typical original force and EMG recordings from the start and end of the fatiguing stimulation in *Exp1* are shown in Figure 1A. This intense stimulation protocol (3 s on, 1 s off) resulted in marked decreases in tetanic force (~55%) and even larger decreases in superimposed (~80%) and potentiated (~70%) twitch forces (Figure 1A). Moreover, the M-wave properties were severely affected with the amplitude being decreased by ~65% and the latency increased by ~7% at the end of the stimulation period (Figure 1B). Thus, these intermittent contractions resulted in action potential impairments, which can contribute to the reduction in superimposed twitch force during fatigue.

#### Exp2

**Figure 2:**
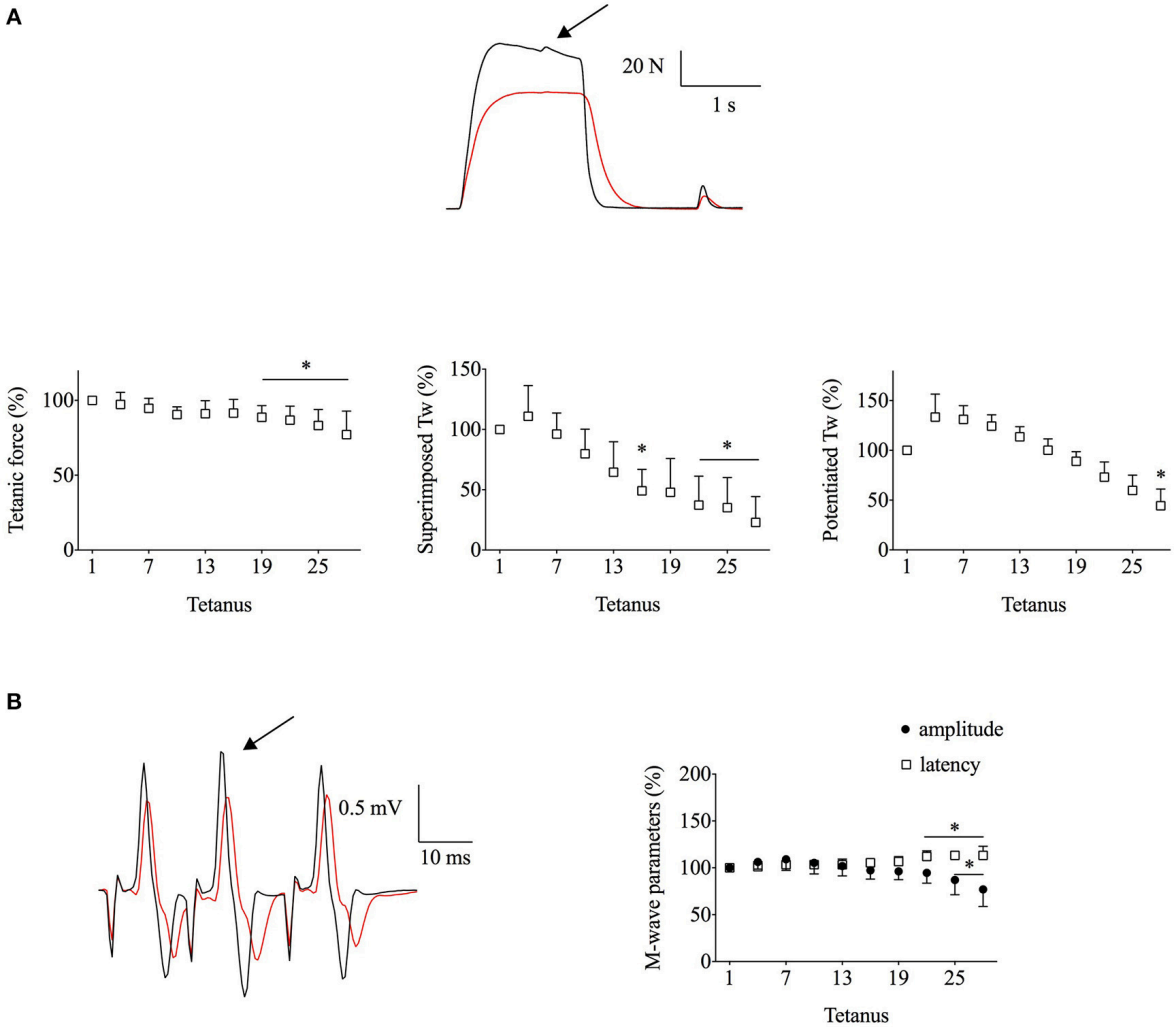
**The less intense stimulation with ischemia in ***Exp2*** resulted in marked reductions in superimposed twitch (Tw) force despite only minor changes in M-wave properties**. Human *adductor pollicis* muscles were stimulated at 30 Hz, 1.5 s on–3 s off. **(A)**, the upper part shows representative force records of the first and last fatiguing contractions and the lower part mean data of tetanic and peak twitch forces; expanded superimposed twitches are shown above the tetanic force records. **(B)**, EMG records (left) and mean M-wave amplitude and latency (right). Black and red lines in original records correspond to the first and the last tetanus; respectively; arrow indicates the extra stimulation. Data are mean ± SD expressed in percentage of the initial value (*n* = 7). ^*^p < 0.05 shows significant difference from the first tetanus (one-way repeated measures ANOVA).

As action potentials were not preserved in *Exp1*, we used a less intense stimulation protocol in *Exp2* (1.5 s on, 3 s off) in an attempt to preserve action potentials; this low-intensity protocol had to be combined with ischemia to induce a substantial fatigue-induced force loss. Figure 2 shows typical original force and EMG recordings from *Exp2*. At the end of the fatiguing stimulation, the decrease in mean tetanic (~25%) and potentiated twitch (~55%) forces in *Exp2* were smaller than in *Exp1*, whereas the decrease in superimposed twitch force (~80%) was similar (Figure 2A). On the other hand, superimposed M-wave properties were better preserved than during *Exp1* with the mean amplitude being decreased by <25% at the end of the stimulation period (Figure 2B).

#### Exp3

**Figure 3:**
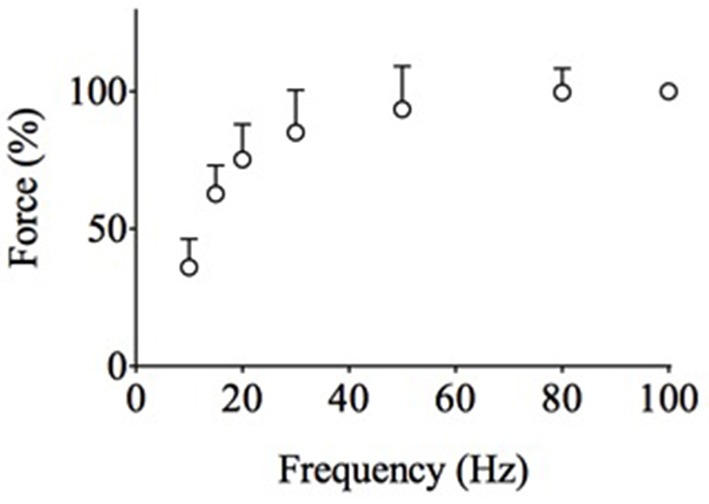
**The force-frequency relationship in unfatigued human ***adductor pollicis*** muscles reveals that 30 Hz is close to maximum force, whereas 20 Hz leaves more room for force to increase in a superimposed twitch**. Data (mean ± SD) collected from *Exp1-3* (*n* = 24). The force at 100 Hz was set to 100% in each subject.

In *Exp1* and *Exp2*, fatigue was induced with 30-Hz contractions. The force-frequency relationship determined before the fatigue run shows that the 30-Hz force was close to the maximum force production of the *adductor pollicis* muscle (Figure 3). This leaves little room for a force increase in superimposed twitches (ceiling effect) and hence the mean superimposed twitch force amounted to only ~5% of the tetanic force (see Table 1). Thus, the severe decrease in superimposed twitch force observed during fatiguing stimulation in *Exp1* and *Exp2* might be due to a fatigue-induced decrease in maximum force (Allen et al., 2008; Place et al., 2010). Accordingly, in Exp2 when the M-wave was better preserved than in Exp1, 100-Hz force was decreased by 35 ± 20% at ~1 min after fatiguing stimulation, which was comparable to the decrease in 30-Hz force. In *Exp3* we therefore induced fatigue with 20-Hz contractions, while leaving other stimulation parameters the same as in *Exp2*. In the unfatigued state, reducing the frequency from 30 to 20 Hz decreased unfatigued tetanic force by ~15% and increased superimposed twitch force by ~45% (see Table 1), which increased the superimposed twitch to tetanic force ratio from ~5% to ~9%.

**Figure 4:**
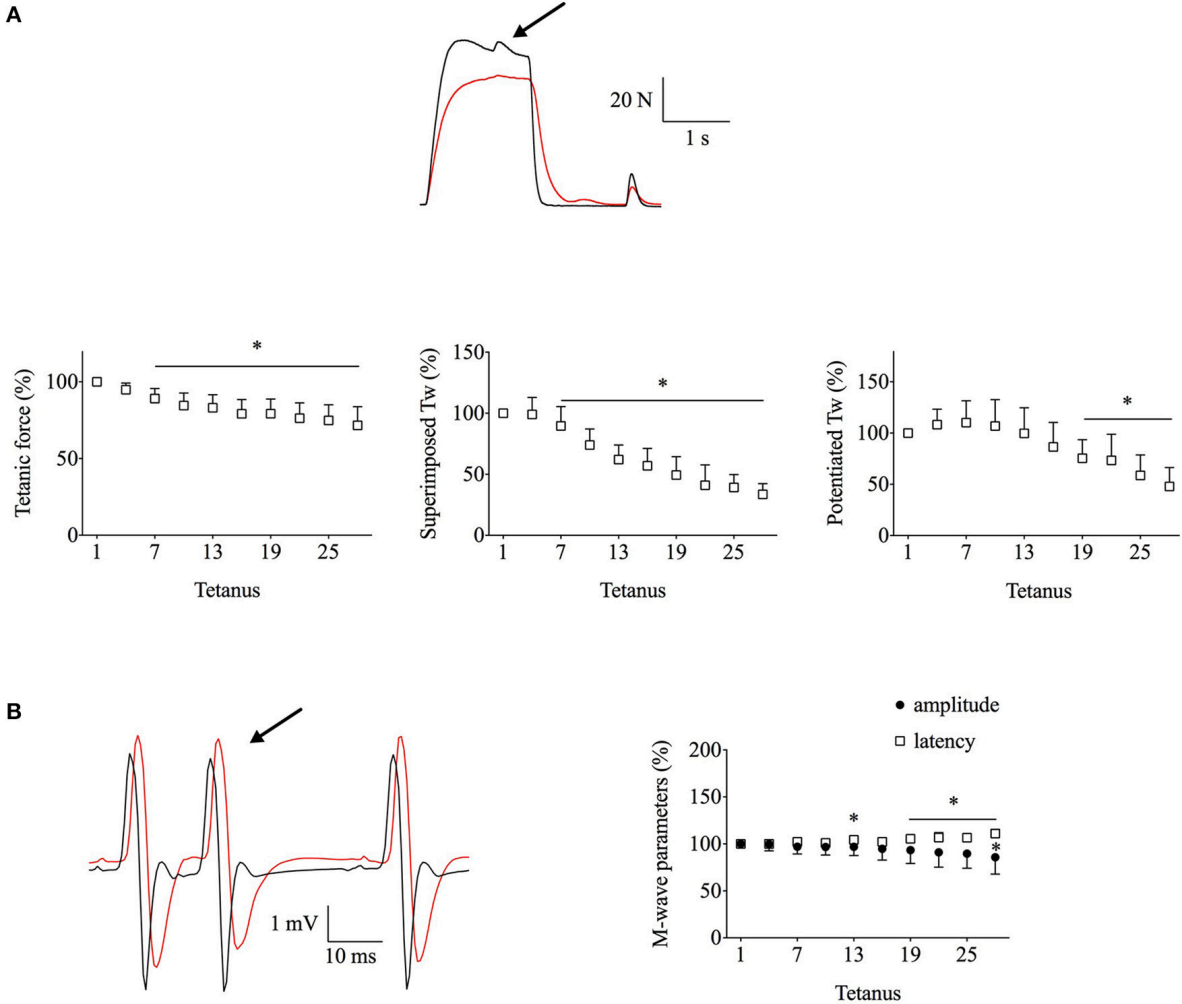
**Superimposed twitch (Tw) force was still markedly decreased in ***Exp3***, which combined the less intense stimulation with ischemia and decreased stimulation frequency during contractions**. Human *adductor pollicis* muscles were stimulated at 20 Hz, 1.5 s on–3 s off. **(A)**, the upper part shows representative force records of the first and last fatiguing contractions and the lower part mean data of tetanic and peak twitch forces; expanded superimposed twitches are shown above the tetanic force records. **(B)**, EMG records (left) and mean M-wave amplitude and latency (right). Black and red lines in original records correspond to the first and the last tetanus; respectively; arrow indicates the extra stimulation. Data are mean ± SD expressed in percentage of the initial value (*n* = 7). ^*^p < 0.05 shows significant difference from the first tetanus (one-way repeated measures ANOVA).

Figure 4A shows that the fatigue-induced force changes in *Exp3* were similar to those observed during *Exp2*, i.e., a major decrease in tetanic and potentiated twitch forces, albeit the superimposed twitch force being somewhat better maintained than in *Exp2* (decreased by ~65 vs. ~80% at the end of fatiguing stimulation). Moreover, M-wave properties were well-maintained with the mean amplitude and latency being decreased by <15% (Figure 4B). Finally, the 100-Hz force after fatigue was reduced by 31 ± 23%, again similar to the decrease in *Exp2*.

### Animal experiments

#### Superimposed and potentiated twitch forces during fatiguing stimulation

**Figure 5:**
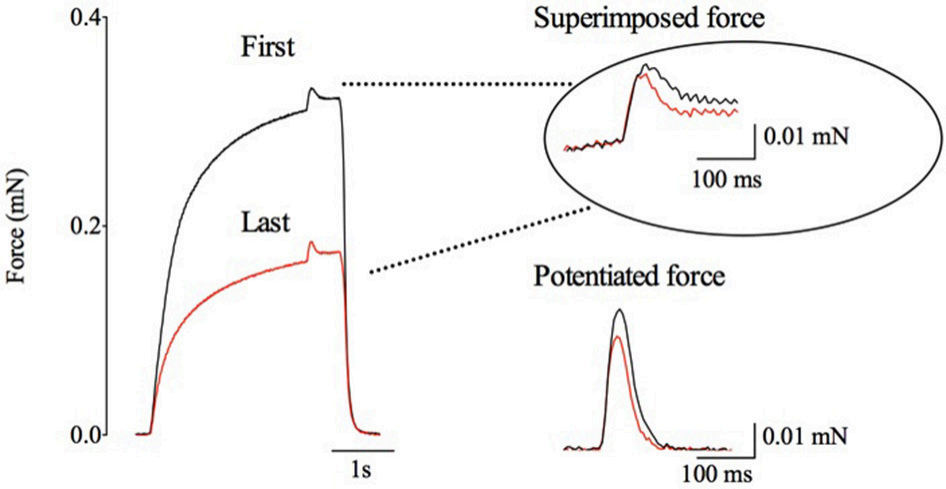
**Typical force records illustrating the relatively small changes in superimposed and potentiated twitch forces in fatigued rat ***soleus*** fibers despite a marked reduction in tetanic force**. Black and red lines correspond to the first and last fatiguing contraction, respectively. Right part shows twitches on an expanded scale. Dotted lines point to the time during the tetanus when the superimposed twitch was elicited.

Rat *soleus* fiber bundles were fatigued with repeated contractions produced at a frequency initially giving 70% of maximum tetanic force, i.e., similar to the relative force achieved with 20-Hz stimulation in the human *adductor pollicis* muscle (see Figure 3). Figure 5 shows typical force traces of the first and last fatiguing contraction in one bundle; right part of the Figure 5 shows expanded traces of the accompanying superimposed and potentiated twitches. Tetanic force was decreased by 51 ± 3% (*n* = 5) at the end of the fatiguing protocol. In contrast to the human muscle results, the superimposed twitch force was well-maintained during fatigue and the mean decrease was actually less than for the potentiated twitch, being 22 ± 15 and 37 ± 28%, respectively.

#### Superimposed twitch with contractile slowing

**Figure 6:**
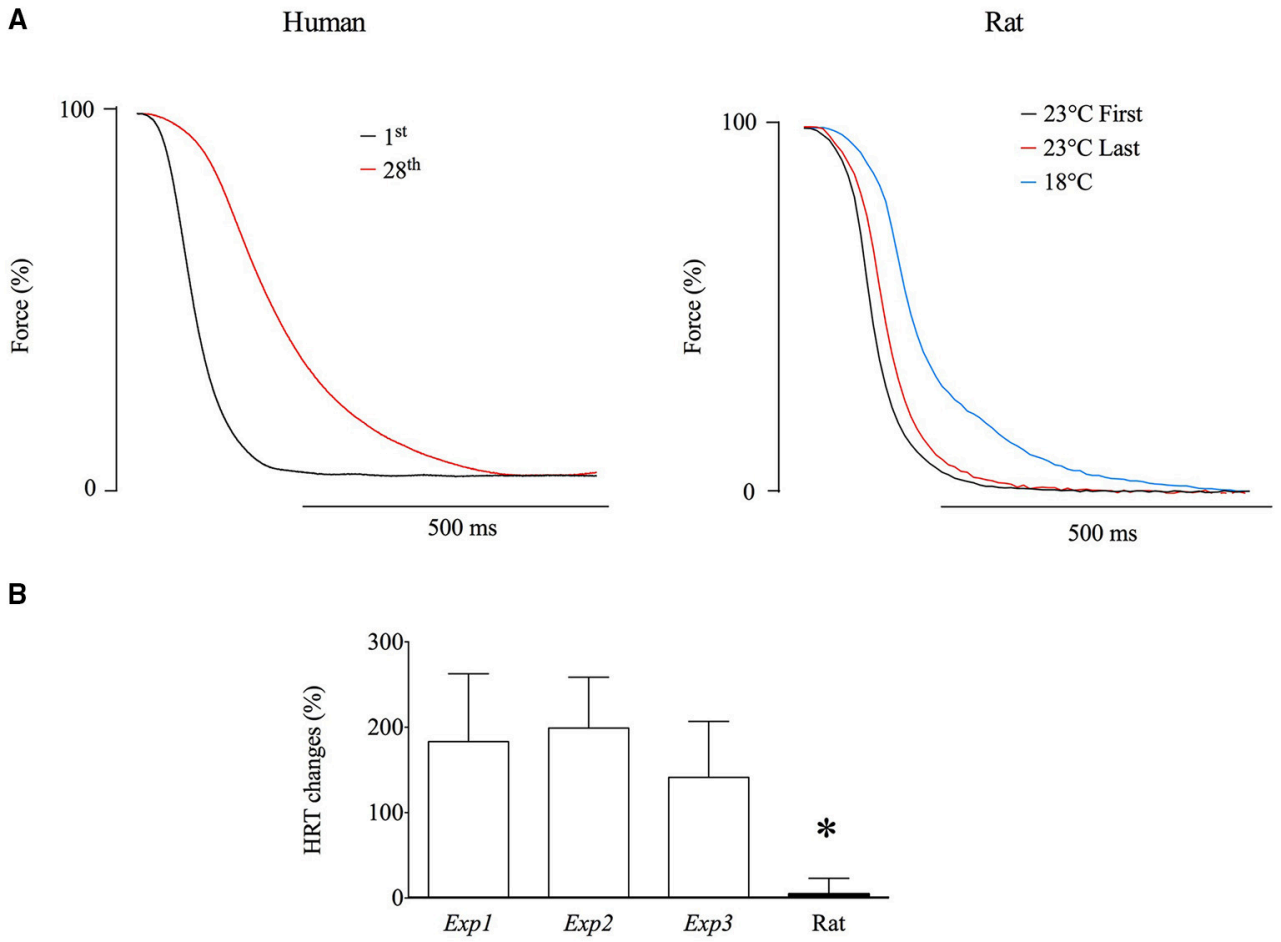
**Repeated tetanic stimulation induced a marked slowing of relaxation in human ***adductor pollicis*** muscles but not in rat ***soleus*** fiber bundles**. **(A)**, representative records of force relaxation after the first (black lines) and last (red lines) fatiguing contractions. **(B)**, mean (± SD) data of the fatigue-induced relative change in half relaxation time (HRT) in *Exp1* (*n* = 10), *Exp2* (*n* = 7), *Exp3* (*n* = 7), and in the rat *soleus* experiments (*n* = 7). ^*^(*p* < 0.05) shows significant difference from human *adductor pollicis* (one-way ANOVA). The slowed relaxation of rat *soleus* bundles at 18°C is illustrated by the blue line in **(A)**; all three force records obtained from the same fiber bundle.

**Figure 7:**
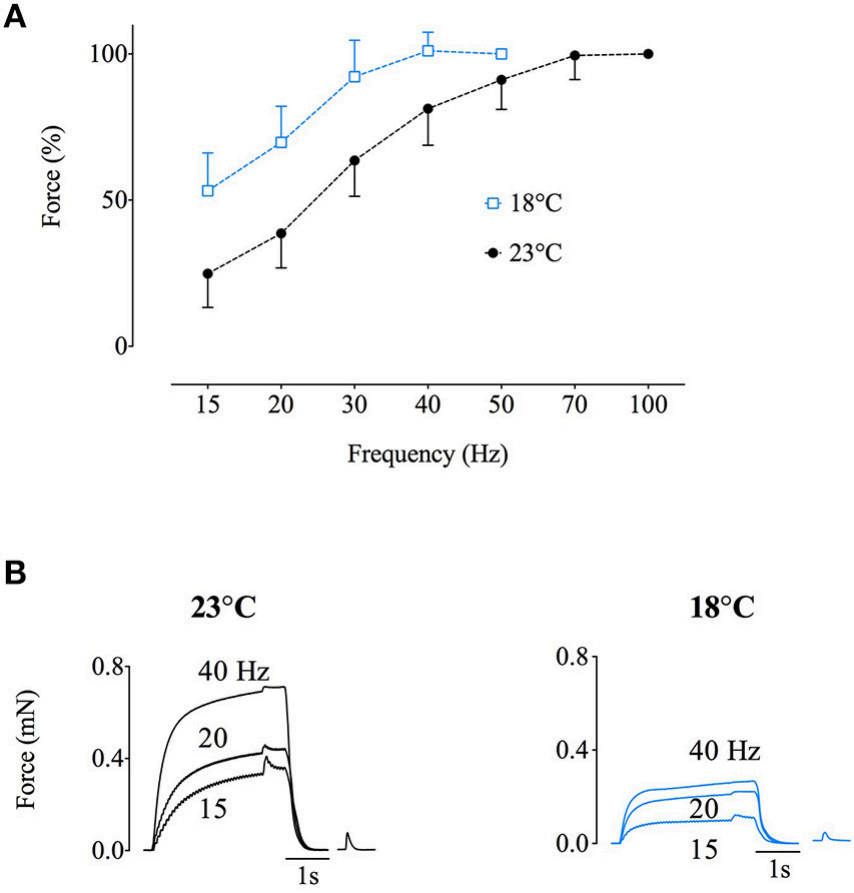
**Slowed relaxation caused a leftward shift in the force-frequency relationship resulting in more fused contractions and decreased superimposed twitch force**. **(A)**, force-frequency relationships obtained at 18°C (blue) and 23°C (black) from rat *soleus* fiber bundles. **(B)**, representative force records at 23°C (left) and 18°C (right) of tetanic stimulations with superimposed twitch at 15, 20, and 40 Hz, and the subsequent potentiated twitch. Data are mean ± SD.

A marked difference between the human and rat muscle experiments was a greater fatigue-induced slowing of tetanic relaxation in the human *adductor pollicis* muscle (Figure 6A). Mean data show a significantly (*p* < 0.05) larger fatigue-induced increase in tetanic half-relaxation time (>150%) in the human *Exp1-3* than in the rat *soleus* experiments (~5%; Figure 6B). A slowing of relaxation will increase the fusion in submaximal tetani, which might decrease the superimposed twitch force. To determine whether slowing of relaxation might affect the fatigue-induced decrease in superimposed twitch force observed in the human *adductor pollicis* muscle, rat *soleus* fiber bundles were cooled by 5°C (from 23°C down to 18°C). This cooling resulted in marked slowing of relaxation of 69 ± 34% (*n* = 7; see Figure 6A). The cooling-induced slowing of relaxation also caused a marked leftward shift of the force-frequency relationship (Figure 7A). The typical force recordings in Figure 7B show that contractile slowing decreased tetanic force and greatly diminished the superimposed twitch force.

## Discussion

In the present study, we used both an *in vivo* human model and an *in vitro* animal model of electrically stimulated muscle to explore peripheral fatigue-induced changes that might affect voluntary activation as calculated using the ITT. Our results showed a marked reduction in superimposed twitch force following fatigue in the human *adductor pollicis* muscle, even when membrane excitability was relatively well-preserved, whereas a small reduction in superimposed twitch force was observed in fatigued rat *soleus* muscle. A pronounced fatigue-induced slowing of relaxation was evident only in the human *adductor pollicis*. When slowing of relaxation was induced by cooling rat *soleus* fibers, we were able to show a marked reduction in superimposed twitch force as observed in the human *adductor pollicis*. These findings highlight that intramuscular factors can confound interpretations of central fatigue when assessed using the ITT.

In *Exp1*, despite the use of intermittent contractions, a large decrease in the amplitude of the superimposed M wave was observed with fatigue. This implicates action potential failure as a mechanism for the decrease in superimposed twitch force and agrees with the results of Gandevia et al. (2013), where human *adductor pollicis* muscles were stimulated continuously. Nevertheless, Gandevia et al. observed decreasing superimposed twitch forces accompanied by increasing M-wave areas during the first minute of continuous 15-Hz stimulation, which implies that failing action potentials may not be the sole cause of the decreased superimposed twitch force.

Earlier studies have shown action potential failure during continuous electrical stimulation (e.g., Bigland-Ritchie et al., 1979; Fuglevand et al., 1993). In contrast, M waves have been reported to be relatively well-preserved after sustained maximal voluntary effort, where the motor unit firing frequency declines as fatigue develops (Bigland-Ritchie et al., 1982, 1983). Thus, our next aim was to assess changes in the superimposed twitch force with fatiguing stimulation protocols where action potentials are preserved. The reduced duty cycle in *Exp2* resulted in better preserved M-wave properties. Yet, the fatigue-induced decrease in superimposed twitch force was similar to that in *Exp1* (~80%, see Figures 1A, 2A), which implies that other mechanism(s) than impaired action potentials contributed to this decrease.

It is possible that the 30-Hz stimulation used in *Exp2* approached the maximum force that the fatigued muscle could produce and hence there was little room for a force increase with the extra stimulation pulse. The stimulation frequency was therefore reduced to 20 Hz in *Exp3*. With this lower stimulation frequency, the superimposed twitch force was slightly better preserved than in *Exp2* but it was still decreased by ~65% at the end of fatiguing stimulation. This implies that in the fatigued state, 20–30-Hz stimulation induced close to maximum myofibrillar force production. The reduction in maximum force in *Exp2* can be estimated as: 30-Hz stimulation gave ~85% of the maximum force in the unfatigued state (see Figure 3) and the 30-Hz force in fatigue was ~75% of the initial (see Figure 4A), which gives a fatigue-induced reduction of maximum force to ~65% (0.85 × 0.75) of the initial. Interestingly, this estimation arrives at the same value as the mean reduction in 100-Hz force measured after the fatiguing stimulation in *Exp2*. Thus, these results indicate that the force of fully Ca^2+^-activated myofibrils was decreased by ~35% in the fatigued state. This reduction in myofibrillar force production is most likely a consequence of metabolite accumulation (Westerblad et al., 2002; Cairns, 2006; Allen et al., 2008; Kent-Braun et al., 2012).

We previously showed a relative increase in the superimposed twitch force during fatigue induced by repeated tetanic stimulation of isolated mouse FDB fibers (Place et al., 2008) which, had it been observed during voluntary contraction in humans, might have been interpreted as decreased voluntary activation. These results differ from those obtained with electrical stimulation of human *adductor pollicis* muscle by Gandevia et al. (2013) and in the present *Exp1-3*. This difference might be due to mouse FDB fibers being fast-twitch (Calderón et al., 2009) and human *adductor pollicis* muscle containing mainly slow-twitch fibers (Johnson et al., 1973). Here we performed experiments on isolated fiber bundles of slow-twitch rat *soleus* muscles (Mizunoya et al., 2014; Soukup and Diallo, 2015) and obtained results similar to those with mouse FDB fibers, i.e., the superimposed twitch force was relatively spared and hence would also be indicative of a decreased “voluntary activation” during fatiguing stimulation. This implies that during fatiguing stimulation, the rat *soleus* fibers remained on the steep part of the force-frequency and force-Ca^2+^ relationships, where the additional sarcoplasmic reticulum Ca^2+^ release induced by an extra action potential had a large force-enhancing effect (Place et al., 2008, 2010). Thus, the observed differences in superimposed twitch force during fatigue relate to human vs. mouse/rat muscle rather than fast-twitch vs. slow-twitch muscle fibers.

A marked difference between the present findings in human and rat muscle is the greater fatigue-induced slowing of tetanic relaxation in the human muscle, hence leading to increased fusion that leaves less room for a force increase. Accordingly, cooling of rat *soleus* fiber bundles resulted in marked slowing of relaxation, more fused tetani, leftward shift of the force-frequency relationship, and hence a greater decrease in superimposed twitch force than in potentiated twitch force which could be interpreted as an increased “voluntary activation,” as observed with human *adductor pollicis* (see above).

Fatigue-induced slowing of relaxation can in principle be due to slowed removal of Ca^2+^ from the myoplasm and/or slowing of the subsequent myofibrillar inactivation, which involves Ca^2+^ dissociation from troponin C followed by cross-bridge detachment (Gordon et al., 2000). Experiments to distinguish between the Ca^2+^ and myofibrillar components showed that the slowing of relaxation in mouse FDB fibers fatigued by repeated tetani was due to the myofibrillar component, whereas the more marked slowing in similarly fatigued *Xenopus* frog fibers was due to both the Ca^2+^ and myofibrillar components (Westerblad et al., 1997). These kinds of experiments remain to be performed during fatiguing stimulation of human muscle. Nevertheless, support for a Ca^2+^ component comes from the fact that the rate of SR Ca^2+^ uptake is slower in human than in rodent muscle fibers (Everts et al., 1989; Lamboley et al., 2013; Reggiani, 2014).

### Limitations

The usage of ITT to assess voluntary activation during maximal voluntary contraction involves complex interactions between peripheral fatigue factors and changes in the pattern of motor unit activation during fatigue. For instance, all motor units are readily activated by supramaximal electrical nerve stimulation, whereas voluntarily later-recruited motor units have been shown to fire at submaximal frequencies even during maximal efforts (Contessa and De Luca, 2013), and consequently their muscle fibers are likely to display only limited peripheral fatigue. Furthermore, fatigue results in decreased motor unit discharge rates. Thus, the stimulation patterns used in the current study cannot completely mimic complex changes in motor unit activation pattern during repeated voluntary contractions. Nonetheless, our findings do support a contribution from intramuscular factors being involved in manipulating evoked forces that are integral to the assessment of central fatigue when using the ITT.

## Conclusions

Measurement of the superimposed twitch force is an essential component when ITT is used to assess the level of voluntary activation. Our results show that peripheral fatigue factors particularly affect the superimposed twitch force, including impaired membrane excitability, decreased myofibrillar force, and fatigue-induced contractile slowing.

## Author contributions

DN, AC, BK, HW, and NP contributed to the conception and design of the study. DN, AC, and NP were responsible for data collection. DN, AC, NB, BK, HW, and NP participated in the analysis and interpretation of the data. All authors were involved in writing the manuscript and approved the final version. All authors agreed to be accountable for all aspects of the work in ensuring that questions related to the accuracy or integrity of any part of the work are appropriately investigated and resolved. All persons designated as authors qualify for authorship, and all those who qualify for authorship are listed. Human experiments were performed in the Institute of Movement Sciences and Sports Medicine of Geneva University, Switzerland and in the Institute of Sport Sciences of the University of Lausanne, Switzerland. All animal experiments were performed at the Cellular Muscle Function Laboratory in the Department of Physiology and Pharmacology, Karolinska Institutet, Stockholm, Sweden.

## Funding

AC and HW acknowledge funding from the Swedish National Centre for Sports Research, and the Swedish Research Council.

### Conflict of interest statement

The authors declare that the research was conducted in the absence of any commercial or financial relationships that could be construed as a potential conflict of interest.
